# Developments in Advance Care Planning in Australia: Potential Opportunities and Roadblocks for an Increasingly Digital World

**DOI:** 10.1007/s11673-024-10396-2

**Published:** 2024-10-23

**Authors:** Casey M. Haining

**Affiliations:** 1 Australian Centre for Health Law Research, Faculty of Business and Law, Queensland University of Technology, 2 George Street, Brisbane City, QLD 4000 Australia; 2 Centre for Health Equity, Melbourne School of Population and Global Health, University of Melbourne, 207 Bouverie Street, Carlton, VIC 3053 Australia

**Keywords:** Advance care planning, Advance care directive, Substitute decision-maker, Digital, Law reform

## Abstract

Australia is committed to looking at ways to modernize its healthcare delivery further by integrating digital health. Advance Care Planning (ACP) is an area of healthcare that would likely benefit from further digitalization. However, while greater integration of technology in the delivery of ACP could help improve practices and lead to increased uptake, the extent to which this is achievable will be influenced, in part, by current approaches to ACP regulation. This article canvasses recent developments and trends in Australian law relevant to ACP and reflects on how these developments may impact the further digitalization of ACP.

Advance care planning (ACP) is a process of planning for future health and personal care whereby a person with decision-making capacity makes their values, beliefs, and preferences known so this can inform medical decision-making at a later time when the person can no longer make or communicate decisions for themselves. In the context of healthcare, ACP as a process is quite broad and can encompass everything from discussions a person may have with their loved ones and care team about their values and preferences to formally documenting such values and preferences within an Advance Care Directive^1^ (ACD) and/or appointing a substitute decision-maker.^2^

The benefits arising from the ACP process are well described. These include increasing the likelihood of a person receiving healthcare that is concordant with their wishes, reducing unnecessary and undesired hospitalization and aggressive treatments, and reducing decisional conflict (Jimenez et al. 2018). While ACP is often considered in the end-of-life context (often in circumstances when a person has a terminal illness and is at risk of losing capacity), ACP may also be beneficial outside such contexts, and indeed, early engagement is often encouraged.

In Australia, the ACP process, like many jurisdictions globally, relies on law to regulate this process to some extent (albeit not exclusively). Indeed, through piecemeal reform, Australian jurisdictions have developed (and subsequently refined) statutory frameworks that govern how medical decisions should be made for people who no longer have the decision-making capacity to make decisions themselves. These modern frameworks are purported to be underpinned by autonomy and have evolved at various paces. In most cases, these frameworks provide for the development of ACDs and the appointment of substitute decision-makers. They also govern the extent to which health practitioners must account for valid ACDs and views expressed by a person’s substitute decision-maker before administering (or withdrawing) certain forms of healthcare to a person who lacks decision-making capacity. Despite the existence of such frameworks to accommodate the ACP process and recent reforms to modernize such regimes further, uptake of ACP (including the completion of ACP documentation) remains limited in Australia (Buck et al. 2021).

Recent technological and artificial intelligence (AI) developments could enhance ACP delivery significantly and increase uptake. The potential for technology to revolutionize the delivery of ACP is reflected in international market trends. Indeed, a recent report suggested that the Advance Directives Market was valued at US$122.74 billion in 2023 and is expected to reach US$588.40 billion by 2032, with high growth expected in the Asia–Pacific region (SNS Insider 2024). Recent technological advancements have been attributed as one of the main driving forces behind such a trend (SNS Insider 2024).

While Australia has yet to fully capitalize on technological advancements to improve the delivery of ACP, this is not to suggest that it may not do so in the future, particularly in light of Australia’s recent commitments to exploring further opportunities to modernizing its delivery of healthcare and integrating digital health (Australian Digital Health Agency 2023). Importantly, any shift towards digitalizing aspects of the ACP process should be considered within the context of existing regulatory frameworks, and reflection should be given to how existing approaches to regulation may support (or hamper) further integration of digital technologies. To this end, this article canvasses some recent developments and trends in Australian law relevant to ACP and reflects on the implications this may have for further digitalizing ACP.

## Trajectory of Reforms

Australia has a complex patchwork of state and territory laws (and policies) that govern how medical treatment decisions should be made for people without decision-making capacity relevant to the ACP context.^3^ While commonality exists, a nationally consistent approach to ACP is lacking. This remains the case despite attempts to harmonize practices across jurisdictions, such as by developing the “National framework for advance care planning documents” (Australian Government Department of Health 2021). This, in turn, is thought to have hampered the uptake of ACP and created uncertainty and confusion amongst health professionals and the community more broadly, particularly with respect to cross-jurisdictional recognition due to different local environments (Australian Government Department of Health 2021).

Over time, state and territory decision-making frameworks have evolved in Australia to give further weight to an individual’s autonomy and reflect modern understandings of the ACP process. This has been achieved by putting a person’s values and preferences at the forefront of decision-making. Consequently, a starting point for clinicians when a person has lost decision-making capacity is to use one’s ACD to guide decision-making, reflecting the fact that they represent the person’s own expression of their values and preferences (albeit expressed previously).

In Australia, ACDs originated in common law. The common law position is that if an ACD is voluntarily made by a competent adult, is clear and unambiguous, and extends to the situation at hand, it must be respected (*Hunter and New England Health Service v A* [2009] NSWSC 761). Today, common law ACDs are still recognized in every jurisdiction except Queensland.^4^

Despite the common law recognition of ACDs, Australian jurisdictions have moved towards affording statutory recognition of ACDs. In 2022, Tasmania became the latest jurisdiction in Australia to introduce a statutory regime for ACDs (see Part 5A of the *Guardianship and Administration Act 1995* (Tas)). Currently, all Australian jurisdictions afford statutory recognition of ACDs except New South Wales.

One of the rationales behind enacting (or modifying existing) legislation in Australia was to codify ACDs in legislation and establish regimes that would regulate the validity and operation of ACDs. To this end, state and territory legislation, to varying extents, now prescribe formality requirements (e.g. patient identifiers,^5^ signature and witnessing requirements, and in some cases, a prescribed form that must be used), specify the circumstances in which ACDs can operate, specify the extent to which ACDs should be relied on, impose obligations on health practitioners to ascertain the existence of ACDs and identity of the person’s substitute decision-maker(s), and offer protections to health practitioners who seek to rely on such documentation when administering (or withdrawing) certain forms of medical treatment.

Initially, statutory ACDs were relatively narrow in scope and, in some cases, remain so. For instance, the Australian Capital Territory’s “Health Direction” only permits a person to make instructions relating to the refusal or withdrawal of medical treatment (*Medical Treatment (Health Directions) Act 2006* (ACT) s 7). Most Australian jurisdictions, however, have now evolved (to varying degrees) to not only include provision for a person to provide consent for particular forms of healthcare but also provide insight into their “values” within their ACD. The notion of “values” is quite broad and captures a wide range of things, ranging from what is important to the person, what they define as quality of life, what health outcomes they deem acceptable, and their cultural values and beliefs.

From a legal perspective, documented “values” do not necessarily have the same binding effect on health practitioners as other forms of instructions due to their lack of specificity. However, they are nonetheless noteworthy, and incorporating values within the same instrument as other preferences has advantages. Indeed, considering a person’s values reflects more contemporary and holistic conceptualizations of the ACP process, which invites a more nuanced reflection of what matters to a person and what they perceive as acceptable medical outcomes. Notably, many regimes still require that consideration be formally given to such values when others are needed to make treatment decisions in relation to a person.

Compared to ACDs documenting values and preferences, legislation has a more significant role in Australia regarding substitute decision-maker appointments, as substitute decision-makers cannot be appointed under common law. Legislation in each jurisdiction now provides for the appointment of a substitute decision-maker (either by the person or through application to a tribunal). Moreover, following recent reforms in the Northern Territory (which took effect in July 2024), each jurisdiction now provides provision for a “default’”form of substitute decision-maker, whereby in the absence of an appointed substitute decision-maker, a person is conferred decision-making authority based on a statutory hierarchy to make decisions on behalf of the person provided they are willing and available (and they are an appropriate person to do so). However, the formality requirements relating to appointment, the scope of authority conferred to substitute decision-makers, and the nature of the statutory hierarchies vary across the states and territories.

The reliance on law to establish frameworks for ACDs and substitute decision-making and govern their operation resembles what has previously been described as a “legal transactional” approach to ACP (Sabatino 2010). The level of prescription and legal formalities prescribed in legislation is intended to reflect the seriousness of such medical decisions, which can even result in a person’s death (e.g. in cases where life-sustaining treatment is withheld or withdrawn). One rationale behind prescribing formalities was so they could act as safeguards and provide evidentiary value. For instance, witnessing requirements are intended to verify an individual’s decision-making capacity and voluntariness to execute the ACD or statutory appointment of a substitute decision-maker. Formality requirements are also thought to help promote recognition of ACDs and compliance amongst the health profession through offering some form of legal protection to health practitioners, who, in good faith, rely on a patient’s ACD which satisfies all the formality requirements, insofar as is permitted by law.

Despite what some might consider a rigid approach to ACP, this is not to suggest that an expression of a person’s values and preferences that do not satisfy statutory requirements (e.g. because they do not comply with formality requirements) will simply be overlooked. Indeed, some flexibility exists within these regimes insofar as such informal expressions of one’s values and preferences can be taken into account indirectly as part of the decision-making process. For example, an informal expression of values and preferences could be used to inform the decisions made by the person’s substitute decision-maker, who must apply a series of decision-making principles when making decisions on behalf of the person. While variation exists across jurisdictions, in general, recent reforms in relevant legislation have shifted towards decision-making principles that are more greatly aligned with human rights principles. Such approaches place a person’s values and preferences at the heart of the decision-making process (including those expressed previously), so where possible these are prioritized, in contrast to more paternalistic and welfare-based approaches such as the “best interests” approach (albeit this approach may still have a role). Hence, even an informal expression of such values and preferences may be incorporated when these decision-making principles are applied. Further, provided they meet the necessary requirements, such documents could be recognized under the common law as an ACD (except in Queensland).

However, while accommodation of such forms of directions and other informal expressions of values and preferences are theoretically possible, there still seems to be a preference to encourage the completion of statutory ACDs (where possible) and retaining formalities in Australia. Indeed, the National ACP Framework, which outlines best practices in relation to ACP, makes it clear that ACDs that satisfy legal requirements should be prioritized over other forms of ACP documents because they are afforded more weight in law (Australian Government Department of Health 2021). Outside of the legal significance that formality requirements have for validity, they have also been found to provide some form of indication of the document’s quality (Buck et al. 2021). Significantly, the absence of specific or a combination of formalities (or quality indicators) could result in a health professional questioning the document’s legitimacy and may lead them to disregard the document’s contents if there is doubt as to whether the document is a true reflection of the person’s values and preferences (Buck et al. 2021).

Australia’s approach differs from some other jurisdictions globally that deliver ACP in the context of healthcare, which either do not have dedicated laws relevant to ACP or have narrow laws and, hence, primarily rely on non-legal documentation for ACP delivery. Singapore is one example of the latter category. Indeed, Singapore has provision for surrogate decision-making and legislation that provides for a form of ACD known as an Advance Medical Directive. However, Advance Medical Directives are narrow in application insofar as they only permit adults to refuse extraordinary life-sustaining medical treatment in cases when the person has become terminally ill. Such narrowness, in addition to cultural factors, has led to a shift in practice in Singapore whereby the use of formal legal ACP documentation has been downplayed and non-legal documentation is given preference, to some extent, as part of a broader ACP programme (Chan 2019).

## Opportunities for Greater Digitalization of ACP

While the preceding section has demonstrated that Australia has progressed in terms of establishing (and subsequently reforming) statutory regimes that provide frameworks that support ACP implementation, as mentioned at the beginning of this article, uptake of ACP remains low in Australia (Buck et al. 2021). Digitalizing aspects of the ACP process and exploring opportunities for AI may enhance the delivery of ACP and could help increase the uptake of ACP amongst Australians. However, the extent to which this can be achieved will be influenced by the extent to which existing legal frameworks will support such implementation. Consideration of this is timely considering recent developments in digital technology and the rise of commercial providers offering digital ACP products.

One obvious way digital technologies can help enhance the delivery of ACP, and has been used to some extent to date, is by using digital storage repositories for a person’s ACP documents so they can easily be retrieved when needed and accessible at the point of care. This is especially important in cases when statutory regimes impose obligations on health practitioners to ascertain the existence of ACP documentation. While there is some evidence to suggest that ACP documents have been stored within electronic medical records, this is far from uniform. Moreover, whether this is possible will be determined by the care setting the person is in. Additionally, while practices may vary, it has also been reported that some health professionals are reluctant to rely on digital copies of certified ACDs (Lacey 2019). Such a finding spurred recent amendments to the South Australian legislation that took effect in March 2024. The amendments resulted in greater clarity around the fact that electronic copies of ACDs could be relied on, provided they were entered into an electronic record system prescribed by regulations (*Advance Care Directives Act 2013* (SA) s 5A*; Advance Care Directives Regulations 2014* (SA) r 4A).

However, even if digital systems exist, depending on the nature of the system, the ability to transfer records as a person moves between care settings may be limited. The rise of digital platforms for storing ACP documentation offered commercially, as well as Australia’s centralized My Health Record system (which permits a person, or another authorized person acting on their behalf, to upload advance care planning information onto the system), have the potential to offer greater portability; however, to date, have been underutilized (Sinclair et al. 2023). Outside issues with underutilization, these repositories lack the safeguarding and auditing mechanisms to monitor uploaded documents for their quality and whether they satisfy jurisdictional requirements (Sinclair et al. 2023). This is significant because, as mentioned above, the statutory regimes that now exist prescribe a series of formality requirements, and without a mechanism to confirm validity prior to uploading, there may be reluctance by health professionals to use such documents when retrieved at the point of care, particularly if there is a doubt with respect to the authenticity of the document (Buck et al. 2021). So, while these repositories do aid retrieval and accessibility, their usefulness is arguably contingent upon the nature and quality of the documents being entered into the system.

Implementing some form of auditing system could address some of the abovementioned issues; however, the differences in requirements across jurisdictions may present challenges. Jurisdiction-based auditing is likely more feasible, which there is some evidence of. Indeed, as part of a state-funded initiative in Queensland, digital infrastructure has been developed, which permits ACP documentation to be uploaded onto the software platform and accessed by health professionals across multiple health settings (Scott et al. 2022). Significantly, every document submitted is audited for quality before being uploaded (Scott et al. 2022). This is especially important in Queensland, where common law ACDs are not recognized. However, such systems are not widespread throughout Australia.

Outside opportunities for improving digital storage, greater integration of digital technology can also facilitate the ACP process. There has been an increasing use of telehealth for ACP discussions. However, formalities imposed by some statutory regimes may hamper the complete digital execution of ACDs and substitute decision-maker appointments. One example is the restrictions some jurisdictions place on using audio-visual witnessing in this context, even if audio-visual witnessing is permissible in the jurisdiction in other contexts.^6^ This means a person could be having an ACP conversation via telehealth, but restrictions on witnessing means the creation of an ACD during that consultation may not be possible. Similarly, some jurisdictions require that ACDs or statutory appointments of a substitute decision-maker are in a prescribed form in order to be considered valid. Accordingly, unless digital platforms used to create documents can account for such requirements (or are otherwise approved for use within the particular jurisdiction), their utility may be limited.

Recent developments in AI also present further opportunities to modernize approaches to ACP and expedite traditional manual processes. Indeed, in addition to assisting with the retrieval of relevant ACP information in relation to a patient, it has been suggested that AI could identify patients who could benefit from end-of-life conversations, as well as be used to manage workflow by sending prompts to clinicians to initiate ACP with patients who are most likely to benefit from it (Chan and Richards 2023). Similarly, it has been suggested that predictive modelling could be applied to predict a patient’s likelihood of consenting to or refusing particular forms of healthcare and, therefore, be used to predict a patient’s preferences and be used to inform medical treatment decisions if the person loses decision-making capacity (Chan and Richards 2023). Using predictive modelling for such a purpose could even eliminate the need for a substitute decision-maker (Chan and Richards 2023). However, the potential utility of any AI will naturally be limited by the nature of its capabilities and the willingness of health professionals to use such technology.

Importantly, some aspects of the ACP process are inherently valuable and are unlikely to be replicated by AI, such as ACP conversations that take place between the person, their loved ones and their care team, which, *inter alia*, can help reduce decisional conflict at later stages. Overreliance on AI may result in some of these aspects of the ACP process being overlooked. Hence, consideration should be given to the potential role of AI in supporting and complementing existing ACP processes, where possible, rather than replacing them.

## Concluding Remarks and Possible Ways Forward

Australia is committed to looking at ways to modernize its healthcare delivery further by integrating digital health (Australian Digital Health Agency 2023). ACP is an area of healthcare that would likely benefit from such integration, which in turn could lead to an increased uptake of ACP in Australia. This article aimed to briefly chart some of the recent developments in laws relevant to ACP in Australia and explore how digital technology could be used in this space. When considered together, there appears to have been some progression towards modernizing ACP approaches, with the potential to further improve delivery, although current regulatory regimes may present challenges.

While it is acknowledged that not all digital implementation is necessarily impacted by current regulatory frameworks, in some cases, it may be. If Australia wants to continue to promote and prioritize legal (particularly statutory) ACP documentation and look to digitalize the delivery of ACP further, then consideration needs to be given to what reform is necessary to facilitate this. While it is outside the ambit of this article to advance an optimal regulatory approach, this article concludes with some possible considerations for Australia if it were to seek to further digitalize ACP practices.

One potential reform option could involve moving away from some rigidities under current frameworks, such as those relating to formality requirements. Indeed, if formality requirements are likely to be challenging to satisfy on a digital platform, they are less likely to be complied with. While, as noted above, there may still be scope for expressed values and preferences that do not constitute an ACD to be used in medical decision-making for a person who has lost capacity, there still seems to be a preference for ACP documentation that meets the legal threshold. However, completely removing such formalities would arguably undermine shifts towards statutory regimes in Australia, which were intended to embed greater safety and increase the likelihood such ACDs are followed.

Accordingly, providing more clarity about the legality and validity of certain digital ACDs and supporting the digital creation of ACDs (including provision for meeting formality requirements digitally, e.g. audio-visual witnessing) through digital platforms (and related system design) may be preferable. Finally, clarification of the role of AI in the context of ACP could also be addressed by regulation. This could include, for example, specific provisions in legislation that outline the extent to which clinicians can rely on AI-generated preferences when making treatment decisions or in the form of professional guidelines that outline best practices.

## Footnotes

1 In this article, the term Advance Care Directive is used throughout for consistency, but the author notes that not all Australian jurisdictions use such terminology. Indeed, other terminology such as Advance Health Directive (Western Australia and Queensland), Advance Personal Plan (Northern Territory), and Health Direction (Australian Capital Territory) is also used.

2 In this article, the term substitute decision-maker is used to refer to any person appointed by a person or tribunal or assumes a substitute decision-maker role as a result of a statutory hierarchy. The author notes various terms are used across the different Australian jurisdictions.

3 There is not scope in this paper, due to the complexities of the laws and the nuances that exist, to provide an in-depth overview or capture all intricacies. Rather the goal of this paper is to canvass general trends in the development of the law in light of recent reforms. For a comprehensive overview of laws related to ACP see Queensland’s University of Technology, End of Life Law in Australia https://end-of-life.qut.edu.au/.

4 Despite s 39 of the *Powers of Attorney Act 1998* (Qld) purporting to preserve the common law, it has been suggested that due to a drafting error in s 66 of the *Guardianship and Administration Act 2000* (Qld) the common law may not be preserved (White and Willmott 2004).

5 Note the term “patient identifiers” is not used in legislation but is used by the author as a catch-all term to describe things such as name, date of birth, and address.

6 For example, in Victoria, s 12 of *Electronic Transactions (Victoria) Act 2000* (Vic) permits witnessing via an audio-visual link. However, as a result of *Electronic Transactions (Victoria) Regulations 2020* (Vic) r 10, this is not permitted in the context of ACDs and substitute decision-maker appointments. In Queensland, temporary laws were in place during COVID-19 following the passing the *Justice Legislation (COVID-19 Emergency Response—Document and Oaths) Regulation 2020* (Qld) r 10 that permitted audio-visual witnessing for the purposes of creating an enduring document (which includes ACDs and substitute decision-maker appointments). However, those provisions have since expired. Despite subsequent legislation making some of the temporary provisions relating to audio-visual witnessing in Queensland permanent, this did not include the provisions relevant to the ACP context.
